# Vaccaria hypaphorine alleviates lipopolysaccharide-induced inflammation via inactivation of NFκB and ERK pathways in Raw 264.7 cells

**DOI:** 10.1186/s12906-017-1635-1

**Published:** 2017-02-20

**Authors:** Haijian Sun, Weiwei Cai, Xu Wang, Yanling Liu, Bao Hou, Xuexue Zhu, Liying Qiu

**Affiliations:** 1 Department of Basic Medicine, Wuxi Medical School, Jiangnan University, Wuxi, Jiangsu 214122 People’s Republic of China; 2 Laboratory of Natural Medicine, School of Pharmaceutical Science, Jiangnan University, Wuxi, Jiangsu China

**Keywords:** COX-2, iNOS, ERK, NFκB, Inflammation, Hypaphorine

## Abstract

**Background:**

Activation of macrophage is involved in many inflammation diseases. Lipopolysaccharide (LPS) is a powerful inflammatory signal contributing to monocytes/macrophages activation associated with increased proinflammatory cytokines expressions. We recently identified that vaccarin was expected to protect endothelial cells from injury. Hypaphorine was abundantly found in vaccaria semen. However, the potential roles and underlying mechanisms of vaccaria hypaphorine on macrophage inflammation have been poorly defined.

**Methods:**

This study was designed to determine the effects of vaccaria hypaphorine on LPS-mediated inflammation in RAW 264.7 cells.

**Results:**

In this study, we demonstrated that vaccaria hypaphorine dramatically ameliorated LPS-induced nitric oxide (NO) release and productions of proinflammatory cytokines including tumor necrosis factor-α (TNF-α), interleukin-1β (IL-1β), IL-6, IL-10, monocyte chemoattractant protein 1 (MCP-1) and prostaglandin E2 (PGE_2_) in RAW 264.7 cells. LPS-stimulated expressions of cyclooxygenase-2 (COX-2) and inducible nitric oxide synthase (iNOS) were down-regulated by vaccaria hypaphorine. Furthermore, vaccaria hypaphorine retarded LPS-induced phosphorylation of ERK, nuclear factor kappa beta (NFκB), NFκB inhibitor IκBα, and IKKβ. Immunofluorescence staining revealed that vaccaria hypaphorine eliminated the nuclear translocation of NFκB in LPS-treated RAW 264.7 cells.

**Conclusion:**

It was seen that vaccaria hypaphorine counteracted inflammation via inhibition of ERK or/and NFκB signaling pathways. Collectively, we concluded that vaccaria hypaphorine can be served as an anti-inflammatory candidate.

**Electronic supplementary material:**

The online version of this article (doi:10.1186/s12906-017-1635-1) contains supplementary material, which is available to authorized users.

## Background

Inflammation is considered as a tissue protective immune response against injurious stimuli including damaged cells, irritants and bacteria [1]. The process of inflammation is regulated by initiating, maintaining and shutting signals [2]. However, the imbalanced inflammation may induce cellular and tissue damage in different diseases such as atherosclerosis [3], hypertension [4], diabetes [5], cancer [6], and neurodegenerative disorders [7].

The immune system may produce inflammation mediators response to chemical, physical, or infectious stress [1]. Macrophages are critical determinants for multiple biological processes during the immune response [7]. Macrophages can release various cytokines and growth factors to exert three major functions including antigen presentation, phagocytosis, and immunomodulation during the process of inflammation [8]. Injured or activated macrophages may coordinate inflammatory responses through releasing various inflammatory mediators [8]. Overproduction of pro-inflammatory cytokine by macrophages leads to destructive inflammation in the body [9].

Lipopolysaccharide (LPS) acts as a switch for macrophages activation as evidenced by excessive expressions in nitric oxide (NO), tumor necrosis factor-α (TNF-α), prostaglandin E2 (PGE2), interleukin-1β (IL-1β), IL-6, IL-10, inducible nitric oxide synthase (iNOS), and monocyte chemoattractant protein 1 (MCP-1) [10, 11]. Pro-inflammatory stimuli mediated-upregualtion of cyclooxygenase-2 (COX-2) is a major contributor to PGE2 synthesis [12]. Mitogen-activated protein kinases (MAPKs) and nuclear factor kappa beta (NFκB) signaling pathways may be two important intracellular molecular pathways involving inflammatory cascade response to LPS stimulation in RAW264.7 cells [13–15].

Vaccarin is the main component of *Vaccaria segetalis* seeds [16]. Vaccarin is recently identified to be a major flavonoid glycoside [17]. The emergence of vaccarin has attracted considerable attention due to its diverse biological activities [18]. Vaccarin dose-relatedly promoted the proliferation, migration, tube formation and neovascularization of human microvascular endothelial cells through activation of Akt and ERK signals [19]. The construction of bacterial cellulose-vaccarin membranes exhibited no cytotoxicity for cell growth, which was found to be a potential candidate for wound healing in rat skin models [20]. We recently demonstrated that vaccarin may protect endothelial cells from oxidative stress-induced injury via negatively regulation of Notch signaling [21]. We further established that vaccarin may obviously ameliorate high glucose-mediated endothelial cell injury by reversing cell viability and migratory ability [22]. The existing evidence suggested that vaccarin may function as novel therapeutic agent for endothelium dysfunction. The hypaphorine is an indole alkaloid from *Erythrina velutina* that exhibits sleep promoting effects in normal mice [23]. The hypaphorine from different marine sources is shown to possess anti-inflammatory properties [24]. Fungal auxin antagonist hypaphorine obviously inhibited indole-3-acetic acid-dependent superoxide production by competitively binding to the putative binding site of indole-3-acetic acid [25]. Hypaphorine was also a key component of *Vaccaria segetalis*. However, the potential roles and mechanisms of vaccaria hypaphorine on macrophages inflammation were largely unknown. In this study, we investigated that the possible mechanism by which vaccaria hypaphorine protected RAW 264.7 murine macrophages from LPS-induced inflammation response in vitro.

## Methods

### Drugs and chemicals

Vaccaria hypaphorine was purchased from Shanghai Shifeng technology Co., Ltd.,China. Vaccaria hypaphorine was solubilized in sterile phosphate buffer saline (PBS). The cells including mouse macrophage Raw264.7, human lung adenocarcinoma cell line A549 and mouse lung cancer cell Lewis were purchased from American Type Culture Collection (Rockville, MD, USA). Human microvascular endothelial cells HMEC-1 was obtained from the Health and Medicine Research of French National Institute. Human umbilical vein endothelial cells EA · hy926, mouse fibroblast cells L929, human breast cancer cell line MCF-7 and mouse melanoma cells were gifts from Tang Zhongying Institute of Hematology of Soochow University. Mouse liver cancer cell H22 was a gift from Department of pharmacology, Shanghai Pharmaceutical Industry Research Institute. Cell culture supplies were purchased from Costar (Corning Inc., Cypress, CA, USA). Sulforhodamine B (SRB), MCDB 131, rhodamine B, DAPI, lipopolysaccharide and aspirin (Asp) were purchased from Sigma Chemical Co. (St Louis, MO, USA). RPMI-1640 medium and DMEM were obtained from Hyclone (Logan, UT, U.S.A.). Dexamethasone (Dex) was purchased from Shanghai biological engineering Limited by Share Ltd. Antibody against β-tublin was purchased from Abcam (Cambridge, MA, USA). Antibodies against total or phosphorylated nuclear factor (NF)κB, IκBα, IκB-kinase β (IKKβ) and ERK as well as iNOS, COX-2 were obtained from Cell Signaling Technology (Beverly, MA, USA). The goat anti-rabbit secondary antibody was purchased from SangonBiotech (Shanghai) Co.,Ltd. (Shanghai, China). M-PER Mammalian Protein Extraction Reagent was purchased from Thermo scientific (USA). RNAiso Plus reagent, SYBR® Premix Ex Taq™ and PrimeScript™RT reagent Kit with gDNA Eraser were purchased from Takara Co. (Takara, Otsu, Shiga, Japan). Dex and Asp were served as positive controls.

### Cell culture and treatments

Raw264.7, EA · hy926, L929 and MCF-7 cells were cultured in Dulbecco’s modified Eagle’s medium (DMEM) with 10% fetal bovine serum (FBS, Hyclone, Logan, UT, U.S.A.) and 1 × Antibiotic-Antimycotic Solution. A549, Lewis, B16 and H22 cells were cultured in RPMI-1640 medium supplemented with 10% fetal bovine serum and 1 × Antibiotic-Antimycotic Solution. HMEC-1 cells were cultured in MCDB 131 medium supplemented with 10% fetal bovine serum, 2 mM L-glutamine and 1 × Antibiotic-Antimycotic Solution. These cells were incubated at 37 °C in a humidified air containing 5% CO_2_. The growth medium was replaced every 2–3 day and the cells were seeded onto petridishes or multiwell plates at a ratio of 1–3 upon 80% confluency.

### Assessment of cell viability

The sulforhodamine B (SRB) assay was used to assess the cell viability as our previous report [21]. The cell growth was arrested by incubation of the cells in 2% serum medium for 24 h before treatment. The indicated cells were then seeded in 96-well culture plates at a density of 5 × 10^3^ cells/well, and stimulated with different concentrations of vaccaria hypaphorine (6.25 μM, 12.5 μM, 25 μM, 50 μM, 100 μM, 200 μM) at 37 °C in 5% CO_2_ saturated humidity condition for 24 h. Finally, the optical density (OD) was measured at 540 nm with the aid of a Multiskan MK3 (Labsystem company). The results were expressed as the ratio by normalizing the targeted OD level to that of control OD.

### Determination of nitric oxide (NO) levels

The levels of NO were analyzed using Griess reaction as previously described [26, 27]. Equal volumes of *N*-(1-naphthyl) ethylenediamine and sulfanilic acid were combined to form the Griess reagent. The 100 μl supernatant from each well was collected in all samples, and reacted with 100 μl of the Griess Reagent for 30-min incubation at room temperature. The absorbance was measured at 540 nm. The standard curves were plotted from sodium nitrite standards (ranging from 10 to 60 μM).

### Real-time quantitative PCR analysis

The mRNA levels of tumor necrosis factor-α (TNF-α), interleukin-1β (IL-1β), IL-6, IL-10 and monocyte chemoattractant protein 1 (MCP-1) were detected by a fluorescence quantitative LightCycler 480 Real Time PCR system (Roche, Basel, Sweden) [28]. In brief, total RNA of each sample was extracted by Trizol reagent according to the manufacturer’s instructions. The equal RNA was reverse transcribed to cDNA using PrimeScript™RT reagent Kit with gDNA Eraser (Takara, Otsu, Shiga, Japan). The real-time quantitative PCR was performed in triplicates by using SYBR® Premix Ex Taq™ (Takara, Otsu, Shiga, Japan). The average cycle thresholds (Ct) were employed to quantify fold-change. The relative quantification of gene expression was reported as a relative quantity to the control value by using 2^-△△Ct^ methods. The sequences of primers were listed in the supplemental table (Additional file 1: Table S1).

### Enzyme-linked immunosorbent assay (ELISA) assay

Commercial ELISA kit was used for the measurement of TNF-α,IL-1,IL-6,PGE2 levels (R&D Systems, Inc., Minneapolis, MN, USA) following the manufacturer’s protocols. The cell culture supernatants in each sample were collected and stored -20 °C prior to use. The standards or samples diluent were added into appropriate well of specific antibody pre-coated microtiterplate and incubated for 30 min at 37 °C. The reacted microtiterplate was washed by diluted washing buffer for 5 times. Conjugate was added and incubated for 1 h at 37 °C and then re-washed. The reactions were stopped with 50 μl stop solution, and the absorbance was read at 450 nm with a microtiter plate reader (STNERGY/H4, BioTek, Vermont, USA).

### Western blot analysis

Whole cell lysates were obtained and homogenized by RIPA buffer. The supernatant was collected by centrifugation at 12,000 g for 10 min at 4 °C. Total protein concentration in the homogenate was measured with a protein assay kit (Santa Cruz, Dallas, TX, USA). Total 25 μg of total protein were subjected to SDS-PAGE and electro-transferred onto nitrocellulose membrane (Millipore, USA). The membranes were blocked with 5% nonfat milk for 1 h at room temperature before overnight incubation with indicated primary antibodies at 4 °C. The primary antibodies were as follows: COX-2 (1:1000), iNOS (1:1000), phosphorylation of ERK (1:1000), ERK (1:1000), p-IκBα (1:1000), IκBα (1:1000), p-IKKβ (1:1000), IKKβ (1:1000), p-NFκB (1:1000), NFκB (1:1000), and β-tublin (1:1000). The membranes were washed three times with TBST for 5 min and subsequently incubated with HRP-conjugated secondary antibody. The positive bands were captured using the enhanced chemiluminescent.

### Immunofluorescence microscopy

The stimulated Raw264.7 cells were fixed with 4% formaldehyde for 30 min, and then permeabilized with 0.1% Triton X-100 in PBS for 15 min. After incubation with 10% goat serum for 30 min, and incubated with indicated primary antibody rabbit anti-NFκB overnight at 4 °C. After three washes with PBS, cells were incubated with FITC-labeled secondary antibodies for 30 min. Nuclei were stained with 4’,6-diamidino-2-phenylindole (DAPI) after immunofluorescence staining. Immunofluorescence signals were visualized on a fluorescence microscope (80i, Nikon, Japan).

### Statistical analysis

All results were defined as mean ± S.D.. Comparisons within two groups were made by Student’s *t* test. Statistical analysis was performed by ANOVA/Dunnet *t*-test for multiple group comparisons. A difference of *P* < 0.05 was considered statistically significant.

## Results

### Assessment of vaccaria hypaphorine on cell viability in different cells

The different cell lines RAW264.7, L929, A549, Lewis, H22, B16, MCF-7, HMEC-1 and EA · hy926 were treated with various doses of vaccaria hypaphorine (6.25, 12.5, 25, 50, 100 and 200 μM) for 24 h. SRB assay then showed that vaccaria hypaphorine had no significant effect on the growth of tumor cells and endothelial cells. Vaccaria hypaphorine had no cytotoxicity on these cells including RAW264.7 cells (Additional file 1: Table S2).

### Effects of vaccaria hypaphorine on cell viability response to LPS in RAW264.7 cells

To explore the anti-inflammatory effect of vaccaria hypaphorine, vaccaria hypaphorine was treated on LPS-induced inflammation in RAW 264.7 cells. Firstly, the determination of optical density by SRB assay showed that LPS exerted a slight promotion in RAW264.7 cell proliferation, and combination application of LPS with vaccaria hypaphorine, Dex and Asp had no obvious effect on the proliferation of RAW264.7 cells (Additional file 1: Figure S1).

### Effect of vaccaria hypaphorine on LPS-induced inflammatory responses in RAW264.7 cells

**Fig. 1:**
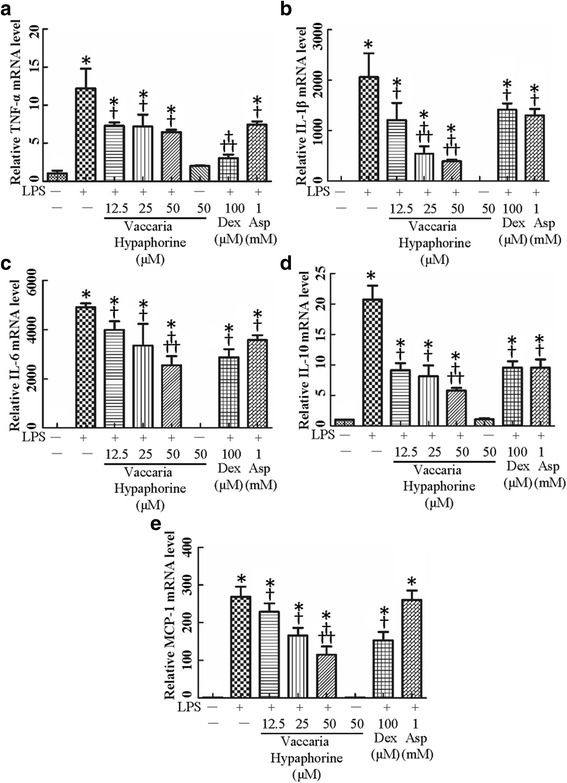
Effects of different doses of Vaccaria hypaphorine (12.5, 25 and 50 μM), Dex (100 μM) and Asp (1 mM) on the mRNA expressions of TNF-α (**a**), IL-1β (**b**), IL-6 (**c**), IL-10 (**d**) and MCP-1(**e**) response to LPS (1 μg/ml)-treated RAW264.7 cells for 24 h in vitro. Values are mean ± S.D. *P < 0.05 vs. Control, †*P* < 0.05 vs. LPS, †† *P* < 0.05 vs. Vaccaria hypaphorine (12.5 μM) + LPS. *n* = 6 for each group. LPS, lipopolysaccharide; Dex, dexamethasone; Asp, aspirin; TNF-α, tumor necrosis factor-α; IL-1β, interleukin-1β; IL-6, interleukin-6; IL-10, interleukin-10; MCP-1, monocyte chemoattractant protein 1

**Fig. 2:**
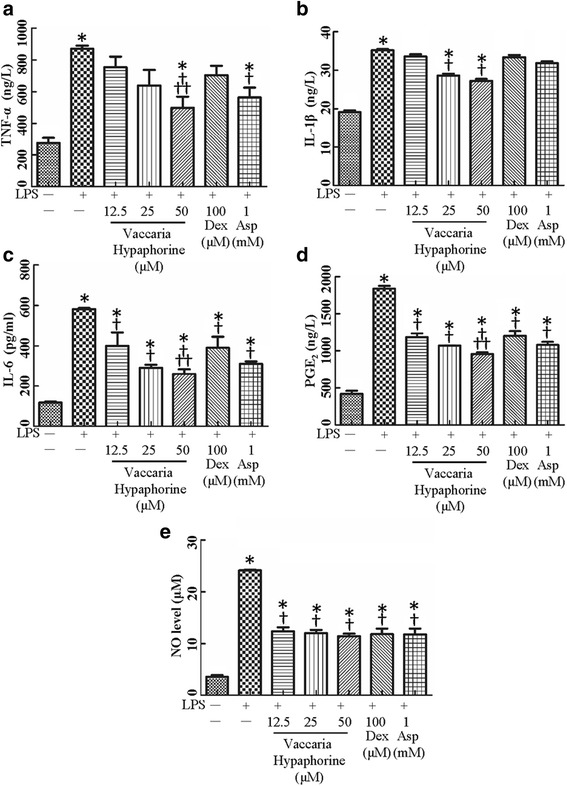
Effects of different doses of Vaccaria hypaphorine (12.5, 25 and 50 μM), Dex (100 μM) and Asp (1 mM) on the TNF-α (**a**), IL-1β (**b**), IL-6 (**c**), PGE_2_ (**d**) expressions and NO production (**e**) response to LPS (1 μg/ml)-treated RAW264.7 cells for 24 h in vitro. Values are mean ± S.D. *P < 0.05 vs. Control, †*P* < 0.05 vs. LPS, †† *P* < 0.05 vs. Vaccaria hypaphorine (12.5 μM) + LPS. *n* = 6 for each group. LPS, lipopolysaccharide; Dex, dexamethasone; Asp, aspirin; TNF-α, tumor necrosis factor-α; IL-1β, interleukin-1β; IL-6, interleukin-6; PGE_2_, prostaglandin E2; NO, nitric oxide

LPS, a Gram-negative bacterial cell wall component, is responsible for pro-inflammatory cytokines accumulation in activated macrophages [29]. Stimulation of RAW 264.7 macrophages with LPS induced remarkable increases in mRNA levels of TNF-α (Fig. 1a), IL-1β (Fig. 1b), IL-6 (Fig. 1c), IL-10 (Fig. 1d) and MCP-1 (Fig. 1e). Vaccaria hypaphorine markedly prevented LPS-induced productions of IL-1β, IL-6, IL-10 and MCP-1 (Fig. 2) in a dose-dependent manner. Vaccaria hypaphorine effectively counteracted TNF-α mRNA levels in LPS-challenged RAW 264.7 cells, but with dose correlation (Fig. 1a). Both Dex and Asp exhibited an inhibitory effect on LPS-induced inflammation reactions at mRNA level in RAW 264.7 macrophages (Fig. 1). In particular, pro-inflammatory cytokine productions were suppressed more effectively by vaccaria hypaphorine (50 μM) than by both Dex and Asp (Fig. 1). Excessive production of NO may act as pro-inflammatory mediators to evoke chronic inflammation contributing tissue damages [30]. Incubation of RAW264.7 macrophages with LPS (1 μg/ml) significantly increased NO overproduction by more than 5 fold, which was effectively reduced by both vaccaria vypaphorine and Dex as well as Asp (Fig. 1f).

Similar to mRNA results, treatment of RAW 264.7 macrophages with LPS caused enormous increases in protein levels of key inflammatory factors including TNF-α (Fig. 2a), IL-1β (Fig. 2b), IL-6 (Fig. 2c) and PGE_2_ (Fig. 2d). The pro-inflammatory effects of LPS were suppressed by vaccaria hypaphorine in a concentration-related fashion. Both Dex and Asp obviously impeded LPS-induced inflammation reactions at protein levels of TNF-α, IL-1β, IL-6 and PGE_2_ in RAW 264.7 macrophages (Fig. 2).

### Effect of vaccaria hypaphorine on LPS-induced COX-2 and iNOS in RAW264.7 cells

**Fig. 3:**
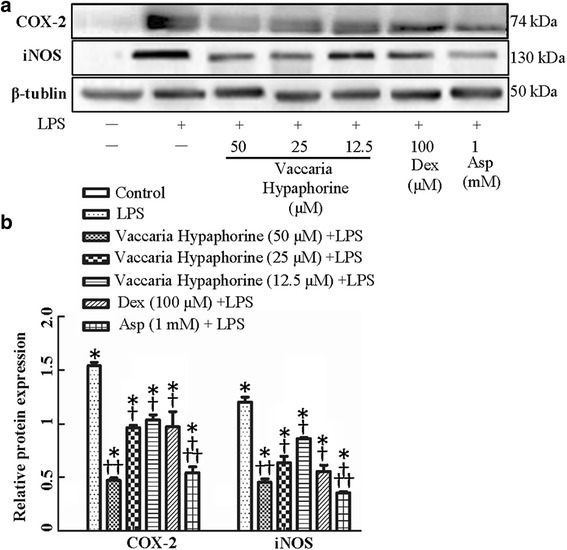
Effects of different doses of Vaccaria hypaphorine (12.5, 25 and 50 μM for 24 h) on the protein expressions of COX-2 or iNOS in response to LPS-stimulated RAW264.7 cells in vitro. **a**, representative images showing effects of pretreatment of different concentrations of Vaccaria hypaphorine (12.5, 25 and 50 μM), Dex (100 μM) and Asp (1 mM) on COX-2 or iNOS levels of RAW264.7 cells response to LPS (1 μg/ml). **b**, quantitative analysis of COX-2 or iNOS protein expressions in different groups. Values are mean ± S.D. *P < 0.05 vs. Control, †*P* < 0.05 vs. LPS, †† *P* < 0.05 vs. Vaccaria hypaphorine (12.5 μM) + LPS. *n* = 6 for each group. NO, nitric oxide; LPS, lipopolysaccharide; Dex, dexamethasone; Asp, aspirin. iNOS, induced nitric oxide (NO); COX-2, cyclooxygenase-2

The iNOS in macrophages may produce excessive NO to exert toxic effects on cells under inflammatory stimuli such as LPS and cytokines [31]. It is accepted that macrophages may induce the expression of inflammatory enzymes such as iNOS and COX-2 during inflammatory responses [32]. The up-regulated iNOS and COX-2 protein expressions were obviously inhibited by vaccaria hypaphorine (Fig. 3). The reduction in NO release by vaccaria hypaphorine may be attributed to iNOS protein inhibition.

### Involvement of ERK or/and NFκB in protective role of vaccaria hypaphorine in LPS-mediated inflammation in RAW264.7 cells

**Fig. 4:**
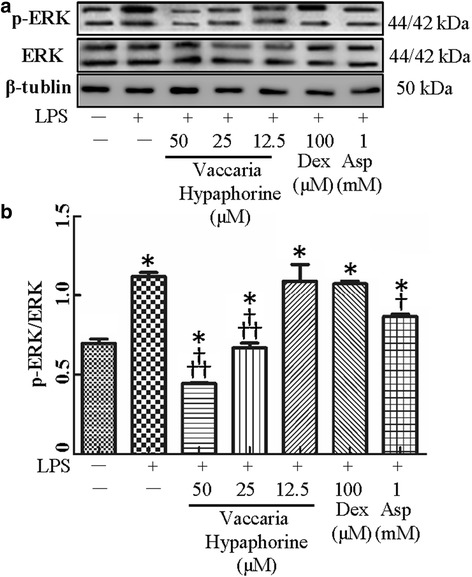
Effects of different doses of Vaccaria hypaphorine (12.5, 25 and 50 μM for 24 h) on the total or phosphorylated protein expressions of ERK in response to LPS-stimulated RAW264.7 cells in vitro. **a**, representative images of Western blot showing effects of pretreatment of different concentrations of Vaccaria hypaphorine (12.5, 25 and 50 μM), Dex (100 μM) and Asp (1 mM) on phosphorylated protein expressions of ERK of RAW264.7 cells response to LPS (1 μg/ml). **b**, quantitative analysis of phosphorylation of ERK. Values are mean ± S.D. *P < 0.05 vs. Control, †*P* < 0.05 vs. LPS, †† *P* < 0.05 vs. Vaccaria hypaphorine (12.5 μM) + LPS. *n* = 6 for each group. LPS, lipopolysaccharide; Dex, dexamethasone; Asp, aspirin

It is well clarified that mitogen-activated protein kinase ERK or NFκB transduction pathways are majorly involved in LPS-induced macrophages inflammation [33]. ERK pathway may be taken as intermediate stage in the regulation of NF-κB activation [34, 35]. Treatment of RAW 264.7 macrophages with LPS promoted the phosphorylation of ERK, which was attenuated by vaccaria hypaphorine at higher dose (Fig. 4), suggesting that the upstream kinases for ERK may be modulated by vaccaria hypaphorine.

**Fig. 5:**
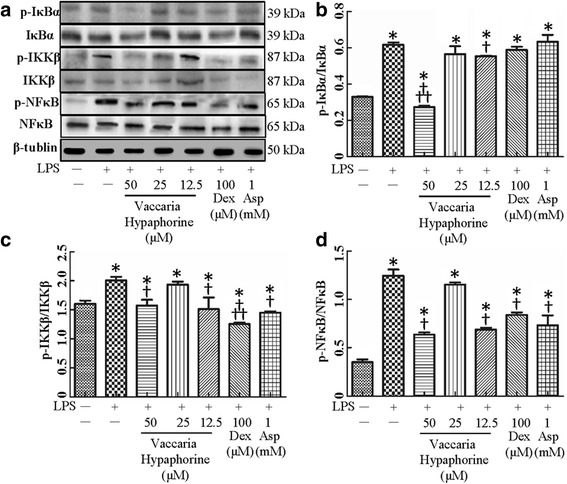
Effects of different doses of Vaccaria hypaphorine (12.5, 25 and 50 μM for 24 h) on the protein expressions of NFκB、IκBα、IKKβ and their phosphorylated protein in response to LPS-stimulated RAW264.7 cells in vitro. **a**, representative images of Western blot showing effects of pretreatment of different concentrations of Vaccaria hypaphorine (12.5, 25 and 50 μM), Dex (100 μM) and Asp (1 mM) on NFκB, IκBα, IKKβ and their phosphorylated protein levels of RAW264.7 cells response to LPS (1 μg/ml). **b**, quantitative analysis of phosphorylation of IκBα. **c**, quantitative analysis of phosphorylation of IKKβ. **d**, quantitative analysis of phosphorylation of NF-κB. Values are mean ± S.D. *P < 0.05 vs. Control, †*P* < 0.05 vs. LPS, †† *P* < 0.05 vs. Vaccaria hypaphorine (12.5 μM) + LPS. *n* = 6 for each group. LPS, lipopolysaccharide; Dex, dexamethasone; Asp, aspirin

**Fig. 6:**
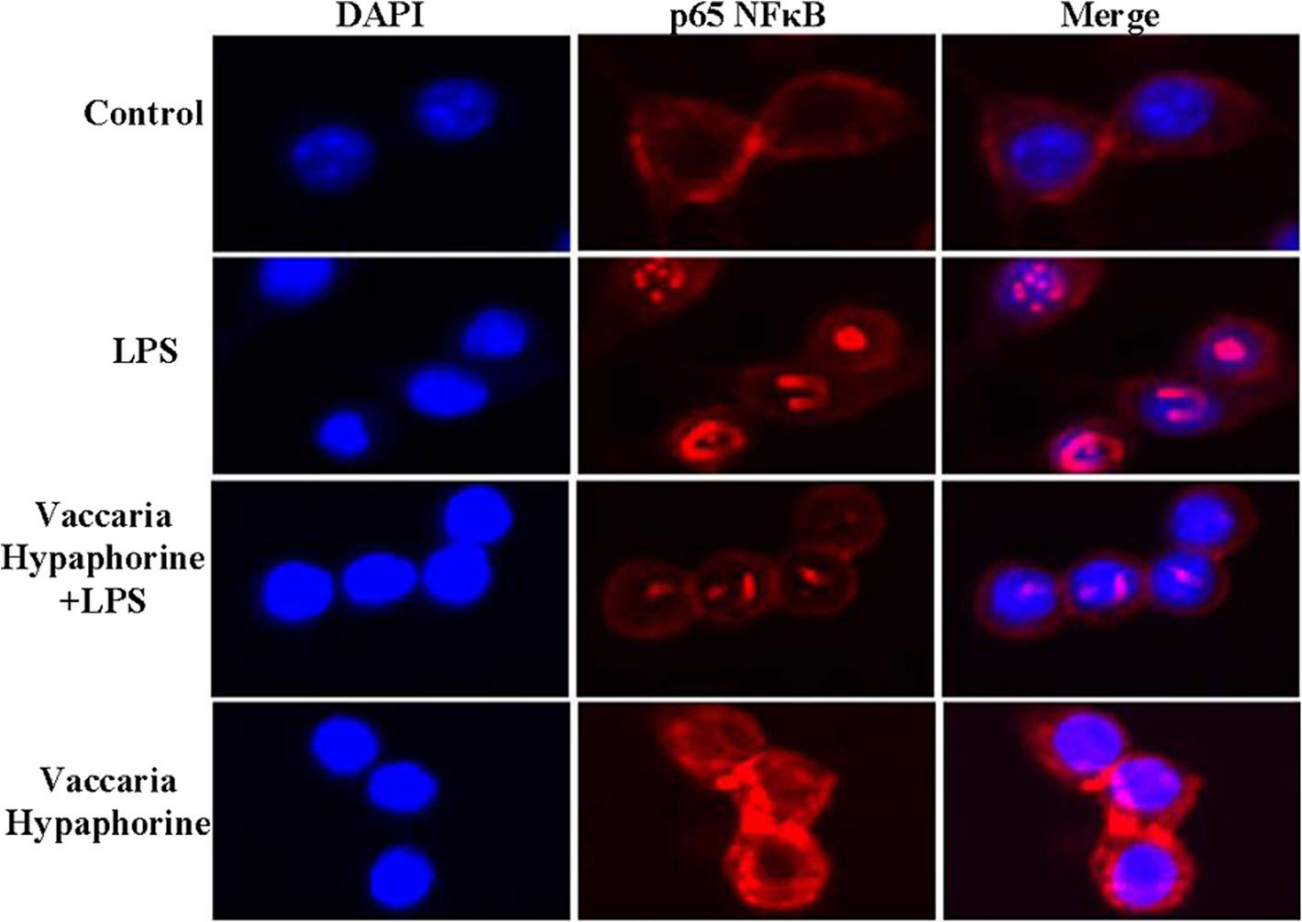
Immunofluorescence staining showing the p65-NFκB distribution in RAW264.7 cells. Nuclei were stained by DAPI (blue). These representative photomicrographs indicated that Vaccaria hypaphorine (50 μM for 24) diminished nuclear translocation of p65-NFκB in LPS(1 μg/ml)-incubated RAW264.7 cells

Nucleus translocation of p65-NFκB is considered as a prerequisite for the transcription [36]. The phosphorylation of IκBα is identified to be upstream events of p65-NFκB translocation. Activation IKKβ is crucial for phosphorylation of IκBα and translocation of NFκB in a canonical pathway [37]. Exposure of RAW 264.7 macrophages to LPS significantly augmented the phosphorylated protein levels of NFκB, IκBα and IKKβ in RAW 264.7 macrophages, whereas vaccaria hypaphorine (50 μM) markedly inhibited the LPS-induced production of phosphorylated IκBα (Fig. 5a), IKKβ (Fig. 5b) and NFκB (Fig. 5c). Furthermore, LPS promoted the nuclear accumulation of p65-NFκB as evidenced by immunofluorescence staining, and which was effectively attenuated by pretreatment with vaccaria hypaphorine (Fig. 6).

## Discussion

Inflammation is an orchestrated and complex event upon a wide range of harmful stimuli such as infection, tissue injury and stimulant [38]. Inflammation may have two paradoxical activities reflected by protection following pernicious stimuli or tissue injury [39]. Dysregulated or misdirected inflammation is a detrimental factor for pathogenesis of many diseases [40]. The complementary and alternative approaches have attracted wide concerns for their protective effects against inflammatory responses in many diseases [41]. In the present study, we explored whether vaccaria hypaphorine exerted a protection from LPS-mediated inflammation in RAW264.7 cells and investigated the underlying mechanism of vaccaria hypaphorine. We identified that vaccaria hypaphorine abrogated inflammatory responses induced by LPS via inhibition of NFκB and ERK signaling pathways.

Natural products including Traditional Chinese herbs have obtained enormous interest for their clinical application value, and they may provide a promising strategy to prevent chronic inflammatory diseases [42]. Macrophages are responsible for initiation, maintenance, and resolution of inflammation [11]. A growing body of experiments demonstrated that activated macrophages may be critical determinants in the development of inflammatory reactions as reflected by overproduction of different pro-inflammatory mediators and cytokines [8]. Mounting evidence demonstrates that LPS acts on toll-like receptor 4 (TLR4) to trigger inflammation cascade in macrophages via excessive productions of TNF-α, IL-1β, IL-6, IL-10 and MCP-1 [43–45]. In this study, we tested whether vaccaria hypaphorine exerted potential anti-inflammatory activity in LPS-challenged RAW 264.7 macrophage cells. Herein, our results showed that the increased mRNA levels of TNF-α, IL-1β, IL-6, IL-10 and MCP-1, and protein expressions of TNF-α, IL-1β, and IL-6 in LPS-treated RAW264.7 cells, these results were in accordance with various published papers [46–50]. It is interesting that vaccaria hypaphorine dose-dependently alleviated mRNA levels of TNF-α, IL-1β, IL-6, IL-10 and MCP-1, but only middle or high dose of vaccaria hypaphorine abolished the protein expressions of TNF-α, IL-1β, and IL-6 in RAW264.7 cells in response to LPS. The different transcriptional or post-translational modifications by various doses of vaccaria hypaphorine may contribute to the differences in mRNA or protein levels of inflammatory mediators in LPS-incubated RAW264.7 cells.

NO is identified to be a pro-inflammatory molecule in the development of various inflammatory diseases. The production of NO is widely taken as a hallmark for macrophage activation, which is indispensable for the pathogenesis of inflammatory diseases [51]. The activated macrophages can stimulate iNOS expressions to generate NO in culture medium. Blockade of iNOS and NO expressions was used to assess the anti-inflammatory potential of many traditional herbs [49]. A few natural products are believed to inhibit the overproduction of NO. Vermelhotin abrogates the iNOS expressions and NO production by selectively inhibiting p38 activation in LPS-stimulated RAW 264.7 macrophage cells [52]. Sterols isolated from *Hericium erinaceum* exerts inhibitory effects against TNF-α and NO production in RAW 264.7 macrophage cells response to LPS [53]. The pseudohypericin, amentoflavone, quercetin, and chlorogenic acid in *Hypericum perforatum* attenuate the PGE2 and NO expressions via activating suppressor of cytokine signaling 3 (SOCS3) in LPS-incubated RAW 264.7 macrophages [54]. In this study, we disclosed that three concentrations of vaccaria hypaphorine had similar inhibitory effect on NO levels, but vaccaria hypaphorine dose-relatedly compromised the increased iNOS expressions in RAW 264.7 macrophages induced by LPS. These results implied that vaccaria hypaphorine was an anti-inflammatory natural product by suppressing NO production in RAW 264.7 cells. COX-2 is largely involved in the synthesis of PGE_2_, which lead to inflammatory symptoms in RAW 264.7 macrophages [55]. We also revealed that vaccaria hypaphorine obviously blocked LPS-upregulated protein expressions of COX-2 and PGE_2_ mRNA levels in a dose-dependent manner. These results hinted that vaccaria hypaphorine may be vital to control immune responses through inhibition of pro-inflammatory cytokines and mediators.

NFκB is a pro-inflammatory transcription factor, which is pivotal for inflammation cytokines deposition and COX2 up-regulation [56]. Several studies have shown that translocation of NFκB from the cytoplasm to the nucleus may be crucial for overexpression of inflammatory mediators such as COX-2, iNOS, TNF-α, IL-1β, IL-6, IL-10 and MCP-1 [57]. The phosphorylation of IκBα and IKKβ is a vital event for NFκB activation [58]. It is established that activation of ERK may be partially responsible for LPS-induced iNOS and COX-2 expressions in RAW 264.7 macrophages [49, 55]. In this study, our results showed that the phosphorylation of ERK, IκBα, IKKβ, NFκB and NFκB nuclear translocation were markedly reversed by vaccaria hypaphorine in LPS-treated RAW 264.7 macrophages. These data indicated that vaccaria hypaphorine may prevent LPS-initiated inflammatory cytokine productions including TNF-α, IL-1β, IL-6, IL-10 and MCP-1, as well as inflammation related enzymes including COX-2 and iNOS in RAW 264.7 cells via inhibition of NFκB and ERK signaling pathways (Additional file 1: Figure S2). Interestingly, our results showed that high dose or low dose of vaccaria hypaphorine counteracted the phosphorylation of IκBα, IKKβ and NF-κB, but vaccaria hypaphorine at middle dose had no effect on phosphorylation of IκBα, IKKβ and NFκB. These unexpected results will be further elucidated in our next researches.

Taken together, our results provide evidence that vaccaria hypaphorine may be proposed as an anti-inflammatory candidate via decreasing the levels of COX-2, iNOS, TNF-*α*, IL-6, IL-10, PGE_2_, and IL-10 and MCP-1. The disruption of NFκB and ERK pathways may be the possible cellular signaling mechanisms whereby vaccaria hypaphorine exerted the anti-inflammatory response in LPS-stimulated RAW264.7 cells.

## Conclusions

For the first time, our results demonstrate that vaccaria hypaphorine plays an essential role in the anti-inflammatory action response to LPS through the inhibition of NFκB and ERK pathways, which may provide a novel therapeutic strategy for treatment of inflammation-associated diseases.

## Additional file

Additional file 1: Table S1. Primer and its parameters of RT-PCR analysis. **Table S2.** Effects of Vaccaria hypaphorine on survival rate of different cell lines. **Figure S1.** Effects of different doses of Vaccaria Hypaphorine (12.5, 25 and 50 μM for 24 h) on the cell survival rate in response to LPS-stimulated RAW264.7 cells in vitro. (A), effects of pretreatment of different concentrations of Vaccaria Hypaphorine (12.5, 25 and 50 μM), Dex (100 μM) and Asp (1 mM) on cell viability of LPS (1 μg/ml)-challenged RAW264.7 cells determined with SRB assay. Values are mean ± S.D. LPS, lipopolysaccharide; Dex, Dexamethasone; Asp, Aspirin; Sulforhodamine B, SRB. **Figure S2.** Schematic indicating the inhibition of vaccaria hypaphorine to alleviate inflammation response by LPS in RAW264.7 cells. (DOCX 391 kb)

## Abbreviations

ASP: Aspirin
COX-2: Cyclooxygenase-2
Dex: Dexamethasone
DMEM: Dulbecco’s modified Eagle’s medium
ELISA: Enzyme-linked immunosorbent assay
IKKβ: IκB-kinase β
IL-1β: Interleukin-1β
iNOS: inducible nitric oxide synthase
LPS: Lipopolysaccharide
MCP-1: Monocyte chemoattractant protein 1
NFκB: Nuclear factor kappa beta
NO: Nitric oxide
PGE_2_: Prostaglandin E2
SRB: Sulforhodamine B
TNF-α: Tumor necrosis factor-α
