# Supplementary material for: Vaccaria hypaphorine alleviates lipopolysaccharide-induced inflammation via inactivation of NFκB and ERK pathways in Raw 264.7 cells
Source: BMC Complement Altern Med. 2017 Feb 20;17:120. doi: 10.1186/s12906-017-1635-1 (PMC5319035; doi:10.1186/s12906-017-1635-1)
Supplement: Additional file 1: Table S1. — Primer and its parameters of RT-PCR analysis. Table S2. Effects of Vaccaria hypaphorine on survival rate of different cell lines. Figure S1. Effects of different doses of Vaccaria Hypaphorine (12.5, 25 and 50 μM for 24 h) on the cell survival rate in response to LPS-stimulated RAW264.7 cells in vitro. (A), effects of pretreatment of different concentrations of Vaccaria Hypaphorine (12.5, 25 and 50 μM), Dex (100 μM) and Asp (1 mM) on cell viability of LPS (1 μg/ml)-challenged RAW264.7 cells determined with SRB assay. Values are mean ± S.D. LPS, lipopolysaccharide; Dex, Dexamethasone; Asp, Aspirin; Sulforhodamine B, SRB. Figure S2. Schematic indicating the inhibition of vaccaria hypaphorine to alleviate inflammation response by LPS in RAW264.7 cells. (DOCX 391 kb) [file 12906_2017_1635_MOESM1_ESM.docx]

**Vaccaria hypaphorine alleviates lipopolysaccharide-induced inflammation via inactivation of NFκB [and ERK pathways](https://www.ncbi.nlm.nih.gov/pubmed/27175331) in Raw 264.7 cells**

Haijian Sun ^1*^, Weiwei Cai ^1*^, Xu Wang ^1*^, Yanling Liu ^2^, Bao Hou^1^, Xuexue Zhu^1^, Liying Qiu ^1&^

1 Department of Basic Medicine, Wuxi Medical School, Jiangnan University, Wuxi, Jiangsu 214122, P.R. China.

2 Laboratory of Natural Medicine，School of Pharmaceutical Science, Jiangnan University, Wuxi, Jiangsu, China.

& Address for correspondence:

Li-Ying Qiu, M.D., Ph.D., Professor, Associate Dean

Department of Basic Medicine, Wuxi Medical School, Jiangnan University, Wuxi, Jiangsu 214122, P.R. China.

Tel: +86-510-85328363

Fax: +86-510-85328605

E-Mail: [qiulydoc@sina.com](mailto:qiulydoc@sina.com)

*These authors contributed equally to this work.

**Table S1 Primer and its parameters of RT-PCR analysis**

| Primers | Sequences (5’-3’) |
| --- | --- |
| GAPDH (Forward) | AGGCCGGTGCTGAGTSTGTC |
| GAPDH (Reverse) | TGCCTGCTTCACCACCTTCT |
| TNF-α (Forward) | CCTGTAGCCCACGTCGTAG |
| TNF-α (Reverse) | GGGAGTAGACAAGGTACAACCC |
| IL-1β (Forward) | GAAATGCCACCTTTTGACAGTG |
| IL-1β (Reverse) | CTGGATGCTCTCATCAGGACA |
| IL-6 (Forward) | TCTATACCACTTCACAAGTCGGA |
| IL-6 (Reverse) | GAATTGCCATTGCACAACTCTTT |
| IL-10 (Forward) | GCTGGACAACATACTGCTAACC |
| IL-10 (Reverse) | CCCAAGTAACCCTTAAAGTCCTG |
| MCP-1 (Forward) | ATCCCAATGAGTAGGCTGGAGAGC |
| MCP-1 (Reverse) | CAGAAGTGCTTGAGGTGGTTGTG |

Note: TNF-α, tumor necrosis factor-α; IL-1β, interleukin-1β; IL-6, interleukin-6; IL-10, interleukin-10; MCP-1, monocyte chemoattractant protein 1; TLR-4, toll-like receptor 4.

**Table S2 Effects of Vaccaria hypaphorine on survival rate of different cell lines**

| Different cells | Relative survival rate of control (%) | | | | | |
| --- | --- | --- | --- | --- | --- | --- |
| Vaccaria Hypaphorine | 6.25 μM | 12.5 μM | 25 μM | 50 μM | 100 μM | 200 μM |
| RAW264.7 | 98.7±0.73 | 98.9±0.68 | 98.67±0.83 | 99.31±0.69 | 99.85±0.50 | 101±0.49 |
| EA·hy926 | 97.1±0.72 | 97.52±0.59 | 97.79±0.31 | 97.37±0.79 | 97.27±0.59 | 98.92±0.71 |
| HMEC-1 | 98.18±0.44 | 99.3±0.28 | 99.21±0.43 | 99.06±0.94 | 99.43±0.46 | 99.63±0.53 |
| L929 | 99±1.13 | 98.54±0.35 | 99.84±0.97 | 98.81±0.37 | 98.71±0.47 | 97.8±0.78 |
| A549 | 97.17±0.06 | 96.69±0.30 | 98.07±0.04 | 97.90±0.74 | 97.02±0.86 | 97.46±0.51 |
| MCF-7 | 97.66±0.54 | 98.67±0.52 | 97.51±0.92 | 98.01±0.43 | 98.2±0.39 | 97.99±0.49 |
| Lewis | 98.62±0.32 | 98.50±0.55 | 98.70±0.72 | 98.16±0.65 | 98.48±0.42 | 98.32±0.82 |
| B16 | 97.31±0.62 | 97.25±0.31 | 97.97±0.59 | 97.57±0.79 | 97.37±0.59 | 98.42±0.54 |
| H22 | 98.1±0.82 | 98.52±0.73 | 98.79±0.31 | 98.37±0.69 | 97.57±0.55 | 98.29±0.46 |

Note: the cell viability was determined with SRB assay, and normalized to the control group.


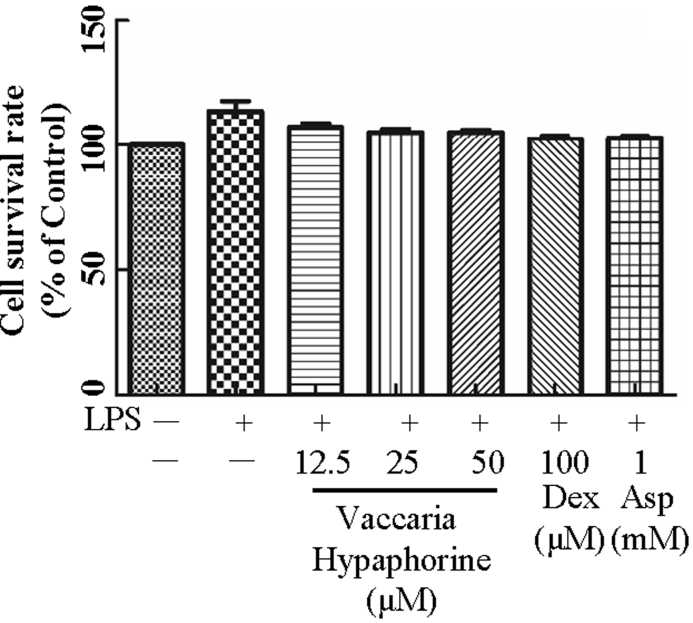


**Figure S1**. Effects of different doses of Vaccaria Hypaphorine (12.5, 25 and 50 μM for 24 h) on the cell survival rate in response to LPS-stimulated RAW264.7 cells *in vitro*. (A), effects of pretreatment of different concentrations of Vaccaria Hypaphorine (12.5, 25 and 50 μM), Dex (100μM) and Asp (1 mM) on cell viability of LPS (1 μg/ml)-challenged RAW264.7 cells determined with SRB assay. Values are mean±S.D. LPS, lipopolysaccharide; Dex, Dexamethasone; Asp, Aspirin; Sulforhodamine B, SRB.


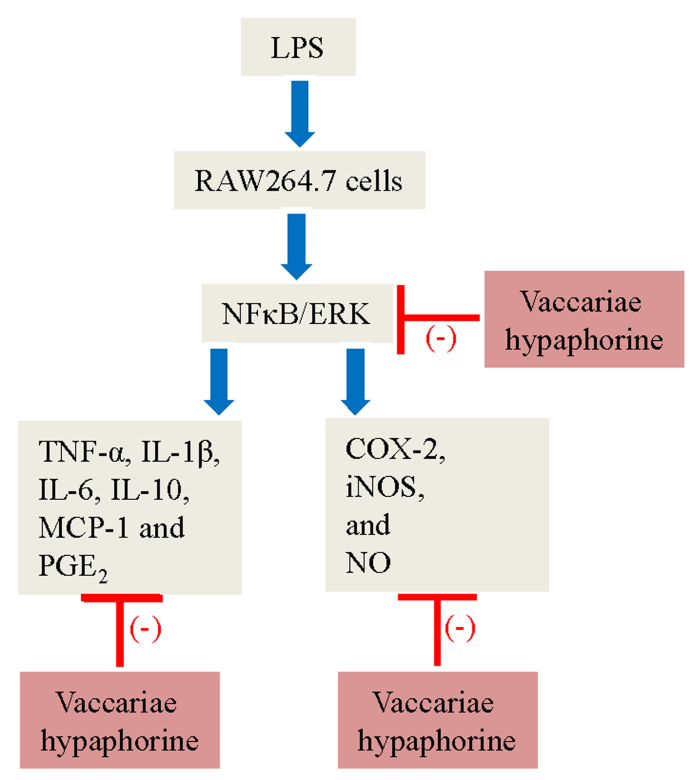


**Figure S2**.Schematic indicating the inhibition of vaccaria hypaphorine to alleviate inflammation response by LPS in RAW264.7 cells.
